# Characterization of Predictive Behavior of a Retina by Mutual Information

**DOI:** 10.3389/fncom.2017.00066

**Published:** 2017-07-20

**Authors:** Kevin Sean Chen, Chun-Chung Chen, C. K. Chan

**Affiliations:** ^1^Institute of Physics, Academia Sinica Taipei, Taiwan; ^2^Department of Life Science, National Taiwan University Taipei, Taiwan; ^3^Department of Physics and Center for Complex Systems, National Central University Chungli, Taiwan

**Keywords:** retina, mutual information, predictive information, omitted stimulus response, stochastic process

## Abstract

Probing a bullfrog retina with spatially uniform light pulses of correlated stochastic intervals, we calculate the mutual information between the spiking output at the ganglion cells measured with multi-electrode array (MEA) and the interval of the stimulus at a time shift later. The time-integrated information from the output about the future stimulus is maximized when the mean interval of the stimulus is within the dynamic range of the well-established anticipative phenomena of omitted-stimulus responses for the retina. The peak position of the mutual information as a function of the time shift is typically negative considering the processing delay of the retina. However, the peak position can become positive for long enough correlation time of the stimulus when the pulse intervals are generated by a Hidden Markovian model (HMM). This is indicative of a predictive behavior of the retina which is possible only when the hidden variable of the HMM can be recovered from the history of the stimulus for a prediction of its future. We verify that stochastic intervals of the same mean, variance, and correlation time do not result in the same predictive behavior of the retina when they are generated by an Ornstein–Uhlenbeck (OU) process, which is strictly Markovian.

## 1. Introduction

The ability to predict or anticipate future events is crucial for the survival of animals. Predicting dynamical inputs can compensate the latency during information transfer and provide predictive information for learning and behavior (Berry et al., 1999; Bialek et al., 2001; Hosoya et al., 2005; Berry and Schwartz, 2011; Leonardo and Meister, 2013). In 2007, Schwartz et al. (Schwartz et al., 2007; Schwartz and Berry, 2008) reported that there will be spontaneous responses from the ganglion cells in the retina of salamanders and mice after a periodic light stimulation is abruptly stopped; with the latency of this spontaneous response being linearly related to the period of the stopped stimulation. In other words, the retina seems to anticipate when the next pulse should have occurred and produce a response if the upcoming pulse is missing. This timed response for the omitted pulse from the retina is known as omitted stimulus response (OSR). Phenomena similar to the OSR have also been reported for induced ocular motor behavior under periodic light stimuli in zebra fish larvae (Sumbre et al., 2008) and growth of slime mold under periodic variation of moisture or temperature (Saigusa et al., 2008).

Ideally, one would like to quantify and model the predictive properties of a retina. Although the phenomenon of OSR has been discovered for more than 10 years, it is still not clear how to relate OSR to the predictive properties of the retina. In OSR, information of the stimulation is apparently coded into the timing of the pulses. However, when there are fluctuations in the inter-pulse intervals of the stimulation, it is difficult to identify or even produce OSR. Therefore, it is not feasible to make use of OSR in inferring the predictive properties of a retina for the general cases of a non-periodic stimulation which should contain much more information than a purely periodic one. Bialek and Tishby have introduced the idea of predictive information based on the statistical properties of the input and output signal of a data processing system (Bialek and Tishby, 1999; Rubin et al., 2016). Recently, this idea has been applied successfully to describe the response of a retina to a stimulation in the form of a stochastic moving bar by computing the mutual information, *I*_*m*_(δ*t*), between the input and output as a function of a time shift δ*t* between the two signals (Palmer et al., 2015). Here, the output at an instant *t* is matched with the input at *t* + δ*t*. And, a negative δ*t* is defined as the time delay of the response of the retina with respect to the input stimulation. Intuitively, the form of *I*_*m*_(δ*t*) should be determined by the predictive dynamics of the retina. However, it is still not clear what kind of information one can extract from *I*_*m*_(δ*t*).

In this work, we report our experimental results in quantifying the predictive properties of a retina by using the predictive information method mentioned above. With a retina plated on top of a multi-electrode array (MEA) and probed by stochastic light pulses, characteristics of *I*_*m*_(δ*t*) is measured as a function of the properties of the light pulses, namely, its mean inter-pulse interval 〈τ〉 and correlation time τ_cor_. Our main finding is that the location of the peak of *I*_*m*_(δ*t*) can be shifted from δ*t* < 0 to δ*t* > 0 by an increase of τ_cor_, suggesting that retina has the ability to predict (with some uncertainties) future events in the stimulation when the stimulation is regular enough. However, this ability of prediction can only be observed when 〈τ〉 is in the range of 100–200 ms, similar to that of the OSR phenomenon mentioned above measured in bullfrog retinas. Furthermore, this predictive property of a retina can be used to distinguish signals generated by an Ornstein–Uhlenbeck (OU) process from those generated by a Hidden Markovian model (HMM), with the signals from the HMM process being identified as more predictable by the retina.

## 2. Materials and methods

Our experiment is similar to that of Schwartz et al. (Schwartz et al., 2007; Schwartz and Berry, 2008) for the study of OSR. The responses of a retina stimulated by spatially uniform light pulses are recorded by an MEA system. The main difference of our experiments with those of the OSR is that the intervals between light pulses are not constant and the stimulation is not stopped abruptly as in the case of OSR. To extend the study of the phenomenon of OSR, we use fluctuating time intervals (with a mean similar to that in OSR) between the light pulses and study the responses from the retina during these stochastic light pulse stimulations. Note that the periodic light intervals used in OSR is a limiting case of this stochastic light interval stimulation when the correlation of the intervals becomes infinite. The followings are the details of the experiments.

### 2.1. Experiment setup

Retinas used in the experiments are obtained under dim red light from bullfrogs which were dark adapted for 1 hour before dissection. A piece of retinal tissue (~2 × 2 mm^2^) is fixed on the MEA by a permeable membrane and perfused with oxygenated Ringer's solution (NaCl 100.0, KCl 2.5, MgCl_2_ 1.6, CaCl_2_ 1.0, NaHCO_3_ 18.0, Glucose 10.0 mM). Each retina preparation can last for 6–8 h for experiments (Ishikane et al., 2005; Xiao et al., 2013). Retinal activities are recorded by MEA with 200 μm inter-electrode distance and 10 μm electrode diameter (MEA60-200-10-PtBlack, Qwane Bioscience). Extracellular potentials from the retina are amplified (MEA1060-Inv-BC, Gain: 1,100, Bandwidth: 1 Hz–3 kHz) and recorded by MC_Rack software at 20 kHz sampling rate. Stimulations to the retina are in the form of a train of stochastic light pulses (pulse duration = 50 ms) generated from an LED (peak of wavelength = 560 nm, intensity = 5 cd/m^2^) which illuminates the whole retina after reflected by a 50%:50% beam splitter. A photodiode (Hamamatsu S1223-01) is placed at the other end of the beam splitter to monitor the stimulation. The intervals between pulses are controlled by a computer to produce a train of pulses with different characteristics which will be described in details below.

### 2.2. Generation of stochastic intervals

Two types of stochastic intervals are used in our experiments. The first type is generated by a HMM following the idea of Palmer et al. (2015), which is associated with a damped harmonic oscillator driven by a noise, with the *i*th intervals being generated as:

(1) $$\tau_{i\;+\;1}=\tau_{i}+v_{i}\Delta$$

(2) $$v_{i\;+\;1}=\left(1-\Gamma \Delta \right)v_{i}-\omega^{2}\tau_{i}\Delta +\xi_{i}\sqrt{D\Delta }$$

where *v* is the rate of change of τ, ξ is a Gaussian noise with zero mean and amplitude *D* = 2. The iteration step size Δ is fixed at 1/60 s. Note that Γ/2ω is kept at 1.06 so that the system is slightly over-damped. To generate the stimulations, a series {τ_*i*_} is first created by the iteration of Equations (1) and (2). Then, the series {τ_*i*_} is rescaled so to have a standard deviation of 20 ms. An offset is also added to {τ_*i*_} to obtain the desired mean 〈τ〉. With this method, the correlation of {τ_*i*_} is not only controlled by Γ. The rescaling of its standard deviation and the addition of offset can also affect the correlation time of the series. The correlation time τ_cor_ of the resultant stimulation must then be measured by computing the decay time of its autocorrelation function. Note that when τ_cor_ tends to ∞, we will recover the periodic stimulation in OSR. With this stochastic pulse train, we can stimulate the retina using temporal patterns with continuously adjustable 〈τ〉 and τ_cor_.

The second type of stochastic intervals is generated by the OU process (Uhlenbeck and Ornstein, 1930), which is a Markovian process that includes a return rate *T* reverting to a mean value in the long run. We construct the OU stimulation as:

(3) $$\tau_{i\;+\;1}=\tau_{i}-\frac{1}{T}\tau_{i}\Delta +\xi_{i}\sqrt{D\Delta }$$

Identical to the HMM stimulation, Δ is fixed as 1/60 s and ξ is the Gaussian noise with zero mean and amplitude *D* = 2. Note that the mean of series {τ_*i*_} returns to zero in Equation (3), so the desired mean 〈τ〉 and standard deviation of {τ_*i*_} (fixed at 20 ms as well) can be adjusted afterwards. Similar to Γ in the HMM stimulation, the correlation time of the OU process can be controlled by *T*.

Stimulations constructed from OU process do not only have “first-order” statistics (mean and standard deviation) similar to the HMM stimulations but also have similar half-life decay of autocorrelation and auto-mutual information. The main difference between the OU process and the HMM is that there is no hidden variable in the OU process. Therefore, any differences in the responses from a retina under these two stimulations may imply that the retina can capture “higher-order” signatures (namely, the hidden variable in HMM) to discriminate between the two processes.

### 2.3. Stimulation protocols

Our experiments consist of recording responses of the retina under stimulations with different characteristics. The protocol is to present each set of stimuli continuously for 5 min in a random order, with an inter-experiment resting time of 2–3 min. All the experiments are carried out in a dark room with temperature around 25 °C. In the results reported below, over ten retina samples are used and at least three retina samples (on average, 10–20 waveforms sorted from each sample) are used to verify each experimental results.

### 2.4. Validity check and data analysis

Responses from the retina are obtained as extracellular potentials from the 60 channels of the MEA system. Spike sorting is performed through the T-Dist E–M sorting algorithm in Offline Sorter software (Xiao et al., 2013). Signals with ambiguous or multiple waveforms are discarded. To verify the proper working of our experimental setup, we reproduce the phenomenon of OSR in our system by following the protocol in Schwartz et al. (Schwartz et al., 2007; Schwartz and Berry, 2008). Briefly, we probe the OSR in the bullfrog retina with periodic stimuli. The peristimulus time histogram from repeated trials of periodic stimuli is obtained and the relative latency of the OSR is measured (Figure S1 in Supplementary Information).

In the experiments reported below, error bars in all the figures reflect the standard deviation between sorted channels. Therefore, the deviation must not be taken as the uncertainties of response from a single recorded channel, which can be quite precise (within 5 ms) in time for OSR. There are strong variations in the recorded responses from different channels of the MEA. As the mutual information between the response recorded by the MEA and the stimulation will be used in this work to quantify the predictive power of a retina, a channel is included for analysis only when its corresponding measurement is significantly (two times) higher than that obtained from its shuffled (time-randomized) version after the bias correction described below. In other words, we exclude channels which record firing patterns that share little information with the stimulation. Less than 25% of the selected units are removed after this validity check. We note that while the deviating performances of the removed channels might signify some different response types, the removal does not affect the conclusion of our statistical tests to be described below. More details of this removal criteria will be given below. Also, because of the finite size of measured data (limited sampling), there will be a bias in the calculation of mutual information. In all mutual information data reported below, the data have been bias corrected by using a method proposed by Strong et al. (1998). Details of this bias correction and the rationale for the choice of other parameters (number of states and bin size) for mutual information computation can be found in the “Information Measurements” Section (Figures S2–S4) of the Supplementary Information, where one can see that the measured mutual information is robust with respect to the choices of parameters. Note that one could also compute the cross-correlogram between the stimulations and the responses of the retina for characterizing its input–output properties. However, as shown in Figure S8 of the Supplementary Information, the cross-correlograms depend strongly on the choice of parameters and their physical meaning for prediction is difficult to interpret.

Finally, to validate our findings, we perform the same experiments on more than five retinas to confirm that they give consistent results with what are reported in the current paper.

## 3. Results

### 3.1. Predictive information for stochastic temporal patterns

**Figure 1:**
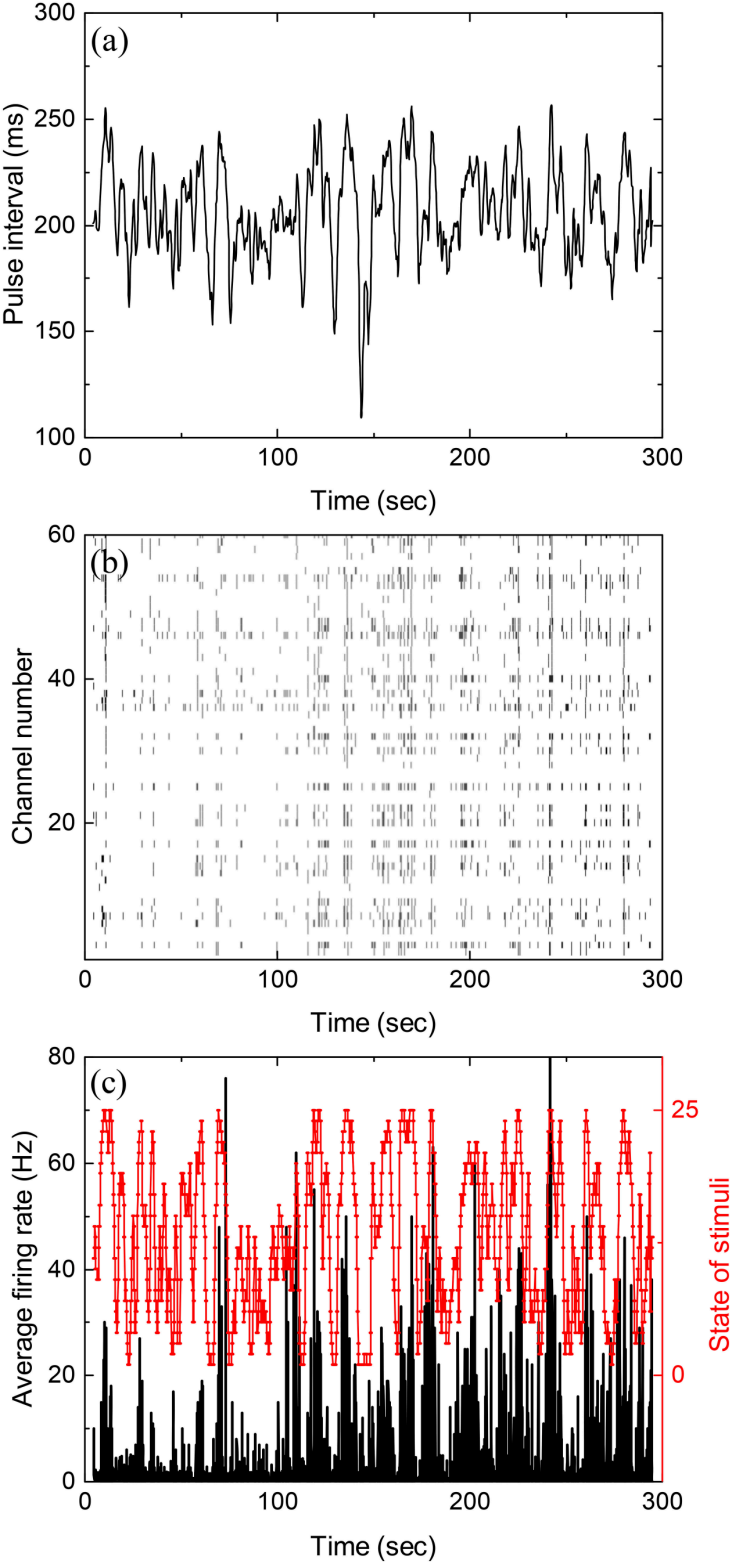
Stochastic pulse intervals and the induced retinal firing patterns. **(a)** Time series of pulse intervals generated by the iteration formula: 〈τ〉 = 200 ms, $\sqrt{\langle {\left(\tau -\langle \tau \rangle \right)}^{2}\rangle }=20$ ms, and τ_cor_ = 2 s. **(b)** Raster plot showing firing timestamps from 60 channels under the input shown in **(a)**. **(c)** Average firing rate of the population recorded in **(b)** with a bin size of 50 ms. To calculate mutual information, the stimuli shown in **(a)** with varying pulse intervals are divided into 25 equally distributed states shown in red. See Figure S5 for the partition of states in Supplementary Information.

Figure 1a shows inter-pulse-interval τ of a typical stochastic pulse train used in the experiments as a function of time (with a discrete time step of 5 ms). The pulse train is characterized by three parameters, namely, the mean inter-pulse interval 〈τ〉, the correlation time τ_cor_ between inter-pulse intervals, and the standard deviation of τ. During each experiment reported below, such a pulse train is presented to the retina for 5 min. Figure 1b is the raster plot for the firings of the retina recorded by the MEA while Figure 1c shows the average firing rate obtained from Figure 1b.

**Figure 2:**
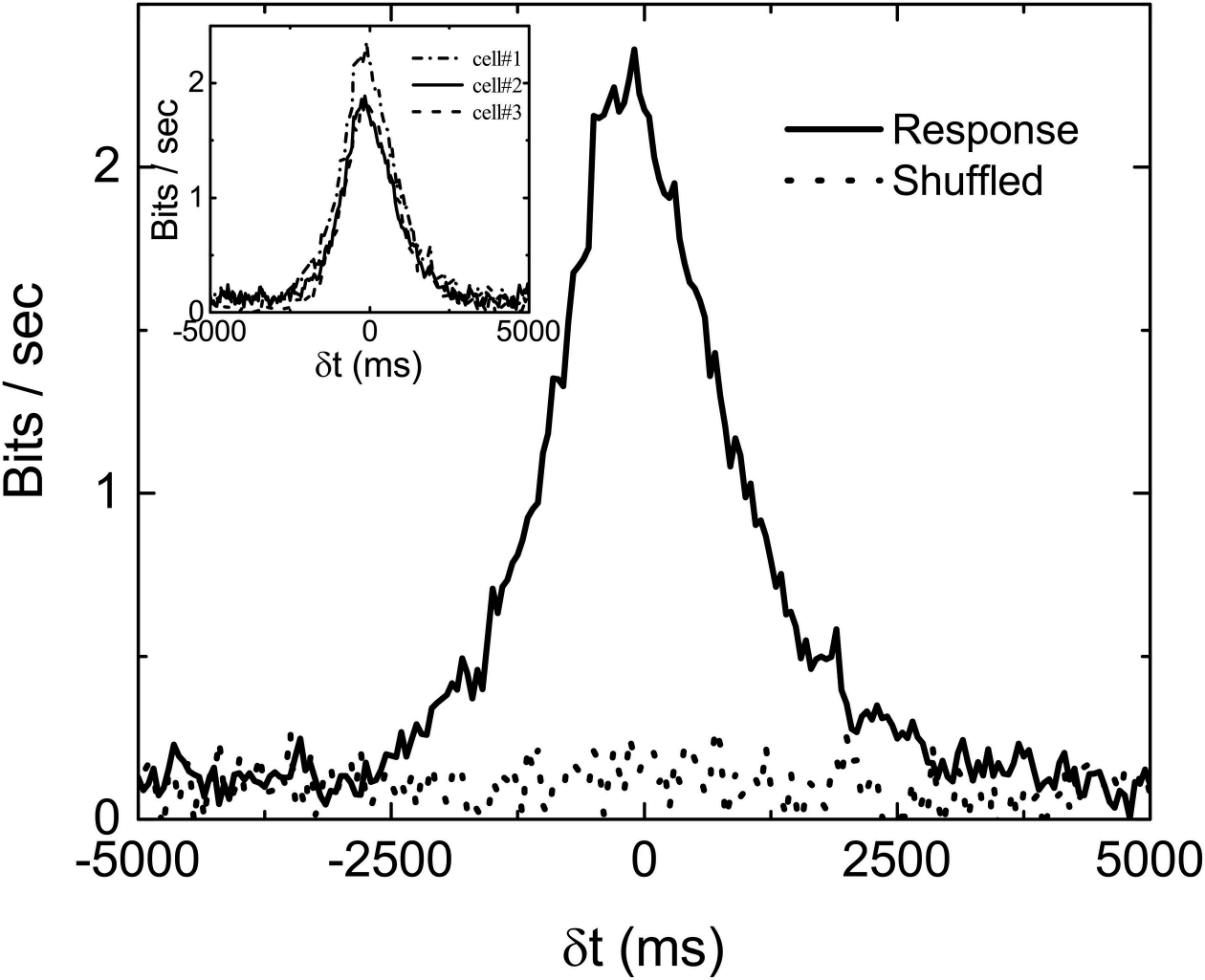
An example of measured *I*_*m*_(δ*t*) with stimulation shown in Figure 1A. $I_{m}^{s}\left(\delta t\right)$ computed from shuffled data is also shown to serve as a base line. Three different *I*_*m*_(δ*t*) obtained from three sorted signals in the same experiment are shown in the inset to demonstrate the variability of the data. The bias due to limited sampling has been corrected for the measured and shuffled data shown here.

Mutual information at different time shift δ*t* between the stimulation (Figure 1a) and response (Figure 1b) can then be calculated by using appropriate binning of the stimulation and response into discrete states. In all the results reported here, the bin size is always 50 ms. Figure 2 is the computed mutual information between stimulation and response from sorted firing waveforms in Figure 1. The interval τ of the stimulation is partitioned into 25 equally distributed states (see Figure S5 in the Supplementary Information for the distribution of states) while the number of spikes in one time window is used as the state index for the response (*R* = {*r*_1_, *r*_2_, …}). The number of states for the response is then the maximum number of spikes for each channel within a time window of 50 ms. The maximum number of spikes within the 50 ms window is typically 10–15 in our recordings. The mutual information at time shift δ*t* is then given by:

(4) $$I_{m}\left(S,R,\delta t\right)=\sum_{i}p\left(s_{i},r_{i-k}\right)\log_{2}\frac{p\left(s_{i},r_{i-k}\right)}{p\left(s_{i}\right)p\left(r_{i-k}\right)}$$

where *p*(*x*_*i*_) is the probability of having a state *x*_*i*_ and *p*(*s*_*i*_, *r*_*i*−*k*_) is the joint probability of the state (*s*_*i*_, *r*_*i*−*k*_). Note that the difference *k* ≡ δ*t*/Δ in time indexes between *s* and *r* denotes a shift in time of δ*t*. It can be seen from Figure 2 that the *I*_*m*_(*S, R*, δ*t*) has a peak located at negative δ*t* and it is non-zero for δ*t* > 0. The location of the peak at negative δ*t* indicates that maximum information is shared between *S* and *R* when *R* lags behind *S*, confirming our intuition that the retina takes some time to reflect/process the information contained in *S* in producing *R*.

Similar to the finding of Palmer et al. (2015), the non-zero value of *I*_*m*_(*S, R*, δ*t*) in Figure 2 for δ*t* > 0 indicates that the firing patterns in retina carry some information on the future events in *S*(*t*) from its history. In fact, *I*_*m*_(*S, R*, δ*t* > 0) is termed predictive information by Bialek and Tishby (1999). It can be seen that *I*_*m*_(*S, R*, δ*t*) is fluctuating around a positive bias below 0.2 bits/s even when δ*t* is much longer than the correlation time of *S*. One would expect *I*_*m*_(*S, R*, δ*t*) to be zero for such a case. This non-physical property of the measured *I*_*m*_ originates from the fact that we are computing *I*_*m*_ from a finite time series. Bias corrections for finite data mentioned earlier have been applied in Figure 2. Without the bias corrections, the bias would have been higher than 0.5 bits/s. It seems that the bias correction can only remove part of the bias due to limited sampling. In order to test whether 0.2 bits/s is the baseline of our measured *I*_*m*_, randomly shuffled data (either states of stimuli or firing rates) are used to compute the mutual information $I_{m}^{s}$. Ideally, the $I_{m}^{s}$ with shuffled data should be zero for all δ*t*. Also shown in the figure is the $I_{m}^{s}\left(\delta t\right)$ curve with shuffled data after bias correction. It can be seen to also fluctuate around 0.2 bits/s, confirming that *I*_*m*_0__ = 0.2 bits/s is the baseline value of our experimentally measured mutual information. As mentioned earlier, not all channels are included for analysis. The criteria is based on the difference between *I*_*m*_(δ*t*) and its shuffled version, $I_{m}^{s}\left(\delta t\right)$ as shown in the figure. If the total area under the curve *I*_*m*_(δ*t*) (−5, 000ms < δ*t* < 5, 000ms) is less than two times of that for $I_{m}^{s}\left(\delta t\right)$, the channel will not be included for analysis.

### 3.2. Measuring predictive power

**Figure 3:**
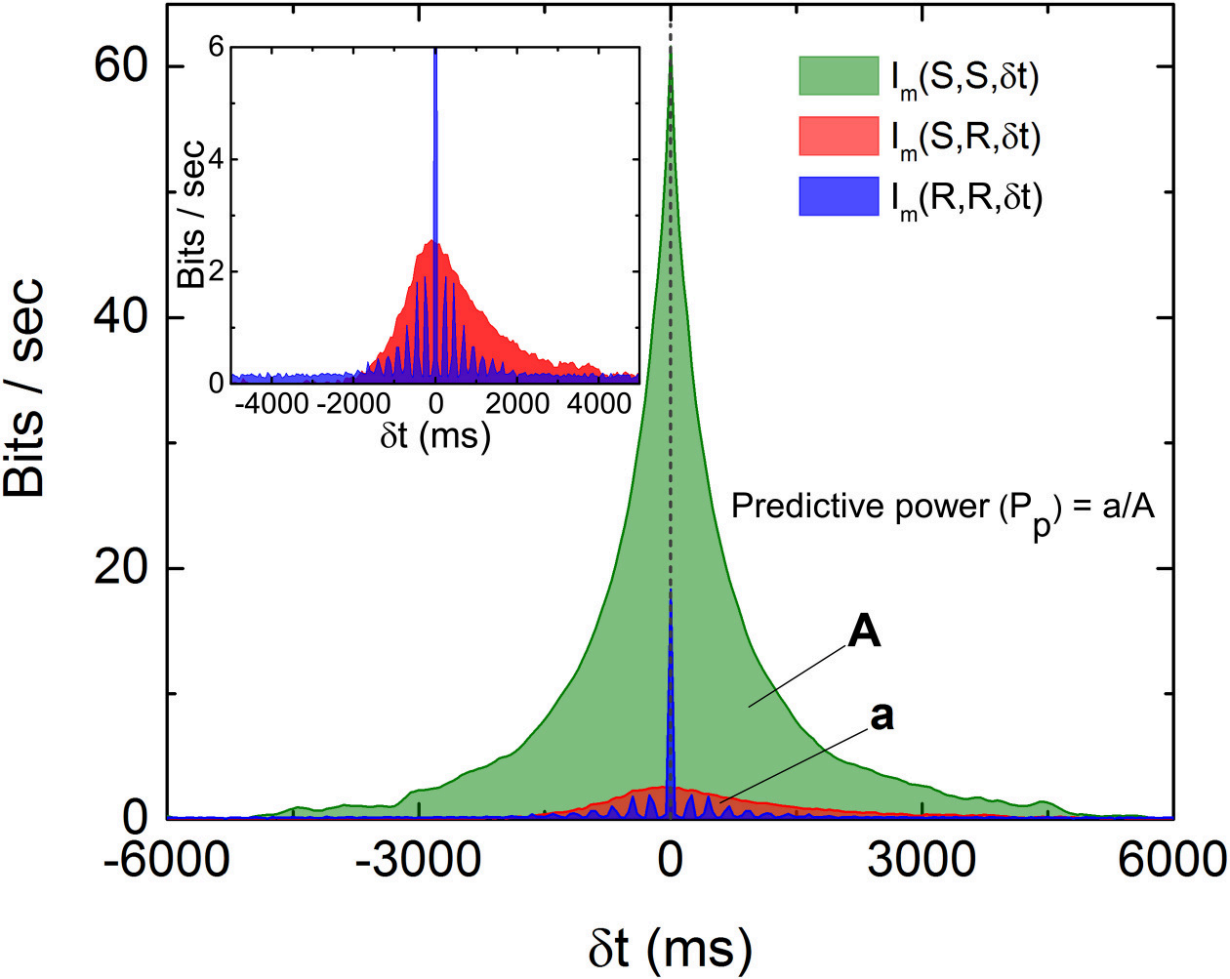
Comparison of the three *I*_*m*_(δ*t*) as described in the text and the definition of predictive power (*P*_*p*_). The areas *A* and *a* as indicated are areas under the curves *I*_*m*_(*S, S*, δ*t*) and *I*_*m*_(*S, R*, δ*t*), respectively, for δ*t* > 0. Note that both *I*_*m*_(*S, S*, δ*t*) and *I*_*m*_(*R, R*, δ*t*) are symmetric about their respective peaks but *I*_*m*_(*S, R*, δ*t*) is not symmetric (inset). The oscillation observed in *I*_*m*_(*R, R*, δ*t*) is caused by the quasi-periodicity of the stimulation light pulses.

To visualize how much information is being shared between *S* and *R*, Figure 3 is a comparison of *I*_*m*_(*S, S*, δ*t*), *I*_*m*_(*R, R*, δ*t*), and *I*_*m*_(*S, R*, δ*t*) from data displayed in Figures 1, 2. It can be seen that only a very small percentage of the information is being shared between *S* and *R*. To quantify the amount of predictive information extracted by the retina, we define the predictive power based on measured *I*_*m*_ as the ratio between the two areas in Figure 3 as *P*_*p*_(*S, R*) = *a*/*A*, where *A* and *a* are the area under the curves *I*_*m*_(*S, S*, δ*t*) and *I*_*m*_(*S, R*, δ*t*) for δ*t* > 0, respectively. This definition satisfies the intuitive notion that *P*_*p*_(*S, S*) or *P*_*p*_(*R, R*) equals to 1, since the predictive power of a signal for itself is fixed as 1, and will allow the comparison of predictive information between different experiments. A remarkable feature of Figure 3 is that while both *I*_*m*_(*S, S*, δ*t*) and *I*_*m*_(*R, R*, δ*t*) decay symmetrically about δ*t* = 0, *I*_*m*_(*S, R*, δ*t*) seems to decay more slowly for δ*t* > 0. Since both *R* and *S* are symmetric with respect to time shift, the asymmetry of *I*_*m*_(*S, R*, δ*t*) possibly comes from the anticipative nature of the retina dynamics in generating *R*. To test whether the conventional linear–nonlinear (LN) model (Chichilnisky, 2001) can capture these special features, we have performed a standard procedure to estimate the firing rate from the stochastic stimulations used in our experiments. Details of the LN model used here can be found in the Supplementary Information (Figures S6, S7). It can be seen that the LN model fails to capture the asymmetry observed in the experiments and over estimates the response delay. As will be shown below, the asymmetry seen in the experiment can be reproduced by a “gedanken” retina which has anticipative power.

### 3.3. Prediction depends on statistics of stimulation

**Figure 4:**
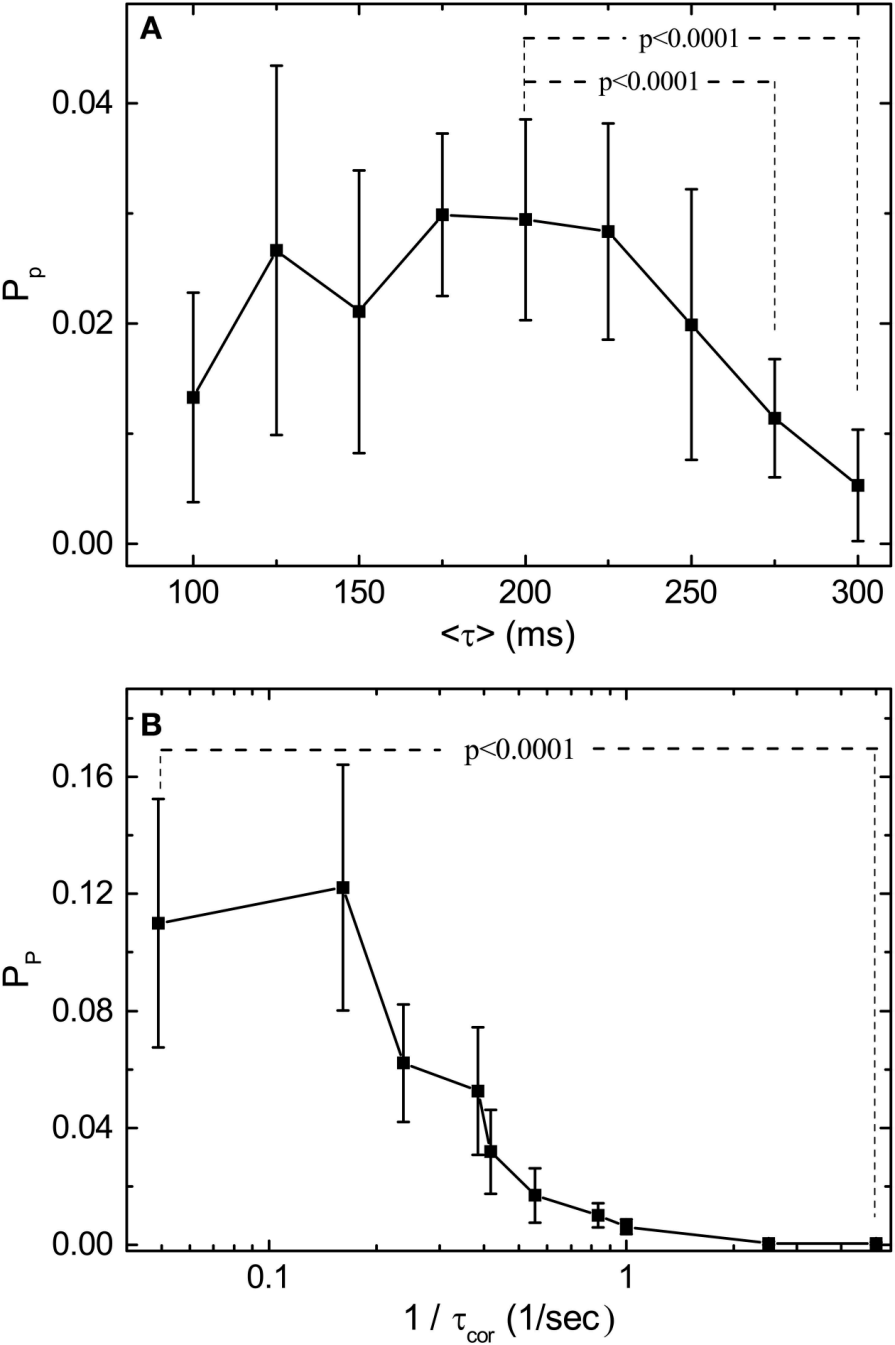
Predictive power (*P*_*p*_) depends on the statistical properties of the simulation light pulses. **(A)** Measured *P*_*p*_ as a function of 〈τ〉 with τ_cor_ = 10〈τ〉 for each 〈τ〉. **(B)** Measured *P*_*p*_ as a function of 1/τ_cor_ with 〈τ〉 fixed at 200 ms. Note that *P*_*p*_ is computed from the mutual information measurements after bias correction. By applying *t*-test, *P*_*p*_ under 〈τ〉 = 200 ms is significantly higher than those under 〈τ〉 = 275 ms and 〈τ〉 = 300 ms. For the effects of τ_cor_, *P*_*p*_ under 1/τ_cor_ = 0.05 is significantly higher than under 1/τ_cor_ = 5. The results are obtained from the same retina, and the error bars indicate the deviation between 17 sorted signals. Specifically, 2 out of 19 channels are excluded after the validity check mentioned in the main text. The deviating performance might signify different response types under stimulation with large τ_cor_. Note that the conclusions of our statistical tests are not affected by this validity check.

With the normalization introduced in Figure 3, we can compare the predictive power *P*_*p*_ for stimulations with various 〈τ〉 and τ_cor_. Figure 4 shows the measured dependence of *P*_*p*_ on 〈τ〉 and τ_cor_ by experiments similar to those shown in Figure 3. Results shown in Figure 4 are obtained from one single retina. The *P*_*p*_ is measured for each channel of the MEA and error bars are obtained from the spread of these measured values. With fixed τ_cor_ = 2 s, it can be seen from Figure 4A that *P*_*p*_ falls off to a very small value around 〈τ〉 = 200–250 ms. Note that a time scale of 200 ms is also the upper limit for a periodic stimulation to produce OSR in the bullfrog retina. Figure 4B shows *P*_*p*_ under stimuli with different τ_cor_ when 〈τ〉 is fixed at 200 ms. Note that the data is plotted in the inverse of τ_cor_. The idea is that the amount of information of the varying pulse interval contained in the time series of the stimulation should increase with the inverse of its correlation time because an purely periodic signal (infinite correlation time) will not contain any information. With this interpretation, Figure 4B indicates that the predictive power of the retina seems to be at its maximum when the information content of the stimulation is low and tends to its minimum when the information content is high. The characteristic time scale (halfway between the max and the min) determined from Figure 4 is when τ_cor_ ≈ 2.5 s.

**Figure 5:**
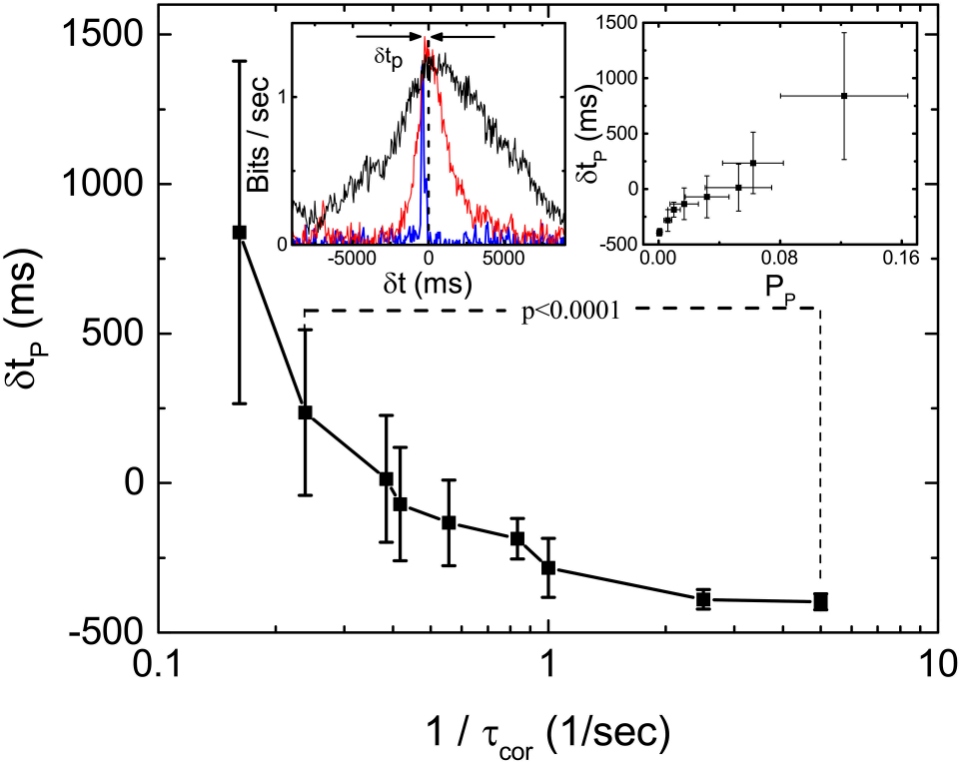
Latency to peak δ*t*_*p*_ of *I*_*m*_(δ*t*) as a function of τ_cor_ obtained from 19 sorted signals in the same retina. The left inset shows the definition of δ*t*_*p*_ and the measured *I*_*m*_(δ*t*) with τ_cor_ = 0.2 (blue), 2 (red), and 4 s (black). Right inset shows the relation between δ*t*_*p*_ and *P*_*p*_ (bias corrected for limited sampling) calculated from the same data. By applying *t*-test, we find that δ*t*_*p*_ is significantly different for 1/τ_cor_ = 0.24 and 1/τ_cor_ = 5.

One interesting feature of the measured *I*_*m*_ during our scan of τ_cor_ at fixed 〈τ〉 is that the peak location of the *I*_*m*_ shifts from negative δ*t* to positive δ*t* as τ_cor_ is increased. Figure 5 shows the dependence of δ*t*_*p*_ as a function of $\tau_{\text{cor}}^{-1}$ where δ*t*_*p*_ is the distance of the peak location of *I*_*m*_ from the line of δ*t* = 0. The inset of Figure 5 shows the definition of peak location δ*t*_*p*_ and the forms of *I*_*m*_(δ*t*) for τ_cor_ = 0.2, 2.0, and 4.0 s. Intuitively, one might expect δ*t*_*p*_ to be always negative because it will always take time for stimulations just to propagate through the different layers and synapses of the retina. That will be true if the retina is just a passive filter. However, if the retina is actively producing anticipative signals for the incoming events, a peak of *I*_*m*_(δ*t*) at δ*t* > 0 can be its signature.

**Figure 6:**
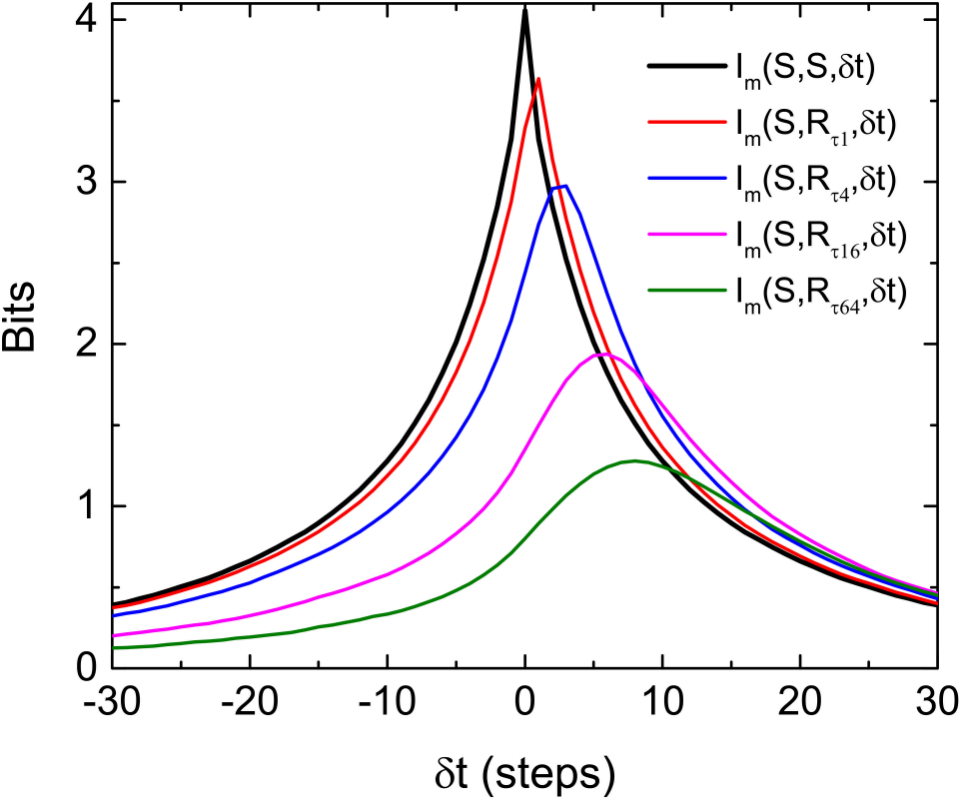
Asymmetry and shift of peak of *I*_*m*_(*S, R*, δ*t*), where *R*s are the responses produced by the “gedanken” retina aiming to estimate a future stimulus. In producing the response *R*_τ*N*_, the “gedanken” retina targets the future that is *N* steps ahead of the current stimulus. The input signal is produced from the same HMM process used in experiments. Note that the peak of *I*_*m*_(*S, R*, δ*t*) moves to the positive time shifts and decreases as the retina attempts to predict further into the future.

To test this later idea, we simulate a situation in which a “gedanken” retina is receiving input from our stochastic pulses at time *t* but then generated response at time *t* by marching Equations (1) and (2) forward *N* steps while using ξ_*i*_ = 0, its most probable value. This gedanken retina is a mathematical construct based on the two equations we used to implement the HMM. Basically, we just pretend that there is an ideally predictive (“gedanken”) retina which can compute the velocity based on the input position. In other words, this “gedanken” retina is anticipating the future of the stochastic input from its present value *N* step ahead by using the velocity information. With this construction of response, we have implicitly assumed that the “gedanken” retina already “learned” the correct parameters of Equations (1) and (2) from it past experience. Figure 6 shows the results of such a simulation with various *N*. It can be seen that the *I*_*m*_(δ*t*) indeed has peaks at positive δ*t*, confirming our intuition that a peak of *I*_*m*_(δ*t*) at positive δ*t* indicates anticipative dynamics of the system. Also, the asymmetry of *I*_*m*_(δ*t*) observed in the experiment is well reproduced here. Note that the shift of the peak is larger when *N* is bigger but the peak value is smaller. That means when the “gedanken” retina is predicting too far into the future, its prediction is less accurate. When comparing our experimental results with different correlation times (Figure 4B) with this simple simulation, it is clear that the real retina is performing prediction. When the incoming signal is more regular (longer correlation time), it can predict deeper into the future.

### 3.4. Interpretation of predictive information

Another remarkable feature of Figure 6 is that the peak value of the *I*_*m*_(*S, R*, δ*t*) from the “gedanken” retina can be higher than that of *I*_*m*_(*S, S*, δ*t*) at the same δ*t*. This means that the “gedanken” retina can have a better prediction about the stimulation in the future than by using the information contained in time series of the stimulation, {*S*_*i*_}. This is because {*S*_*i*_} is produced by an HMM. There is a hidden variable *v*_*i*_. The amount of information contained in {*S*_*i*_} can be smaller than that generated by the “gedanken” retina which knows about both variables by Equations (1) and (2). In other words, prediction is possible in this case because the “gedanken” retina can make use of the hidden variable. If this reasoning is correct, prediction from the retina should not be possible if the stimulations are generated from a Markov process.

**Figure 7:**
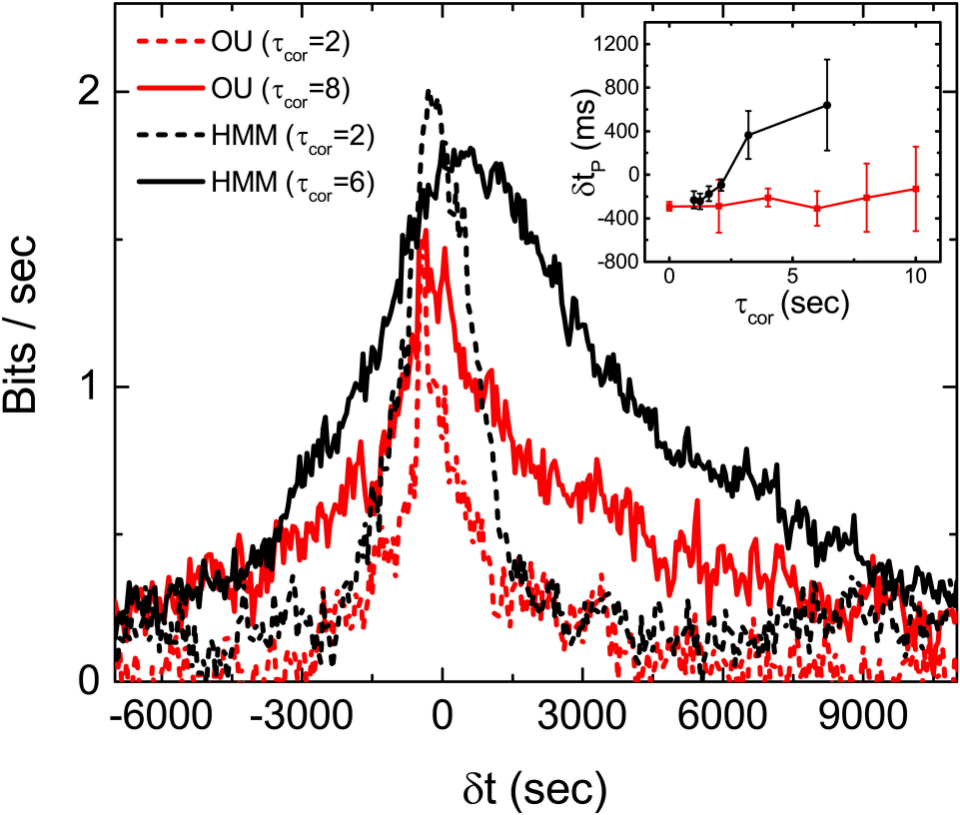
Discriminating OU process and the HMM by a retina. Measured *I*_*m*_(δ*t*) with stimulations generated from an OU process (red) and an HMM (black), each with two different correlation times. Comparison of δ*t*_*p*_ under the two different types of stimulations with varying τ_cor_ is shown in the inset. All measured mutual informations are bias corrected for limited sampling.

Experiments with stimulations generated by an OU process, which is a Markovian process with no hidden variables, are carried out to test this latter idea. To generate the stimulations for the experiments, we tune the OU process in such a way that its time scales and fluctuations are similar to the HMM stimulations used in the experiments reported above. Figure 7 is *I*_*m*_ obtained from the experiments with the OU process for different correlation times. It can be seen that the peaks of *I*_*m*_ from the OU process are all located at δ*t* < 0 and more or less independent of the correlation time of the stimulation. Figure 7 supports the notion that the retina can only perform predictions on an incoming signal with a hidden variable. These results show that the retina somehow manages to make use of this hidden information to anticipate the future time intervals and therefore produce a peak of *I*_*m*_ which is located at δ*t*_*p*_ > 0.

## 4. Discussion

Although the periodic inputs used in OSR and the stochastic pulses used in this study seem to be quite different, the periodic pulses are in fact a limiting case of the stochastic pulses when the correlation time of the inter-pulse intervals becomes infinite. With this consideration, one can think of the periodic pulses used in the phenomenon of OSR as a carrier of information very much like the carrier frequency in an FM radio signal and the information is being encoded into the deviations (fluctuations) of this carrier period. Therefore, the stochastic pulses (with a fixed mean period) used in our experiments are then encoding information in its deviations from the mean. The amount of information encoded can then be characterized by the correlation time: the longer the τ_cor_, the less the amount of encoded information. With a periodic stimulation (infinite correlation time), there is no information encoded. In fact, this carrier wave picture is supported by our finding that both the OSR and the 〈τ〉 for optimal prediction have the same time scale.

We have therefore extended the study of anticipative capability of a retina from probing it with period stimulations to stochastic stimulations. Although the responses of the retina induced by these two types of stimulations seem to be very different, they are of the same nature. In the OSR, a clear transient, spontaneous (anticipative) response can be observed after the termination of the periodic stimulations, while there seems to be no clear anticipative responses can be identified after the termination of the stochastic stimulations. However, the results in Figure 5 show that the retina is generating signals ahead of the stimulation with similar information. In other words, the retina is actively producing spontaneous output corresponding to future events of the stimulation, similar to the case of the OSR. Of course, as we have shown above, prediction is possible only when the incoming signal possesses predictable characteristics such as that generated from a HMM. For signals from the unpredictable OU process, prediction from the retina is impossible. Similar mechanisms of prediction might account for the results reported by Palmer et al. (2015), where the predictive information in a retinal population under a natural scenery input is significantly higher and more long-ranged than those under a random flicking checkerboard.

At first sight, it might seem odd that the response from the retina at present is related to the stimulation at a future time. It should be noted that the future stimulus is not influenced by the output of the retina. There is no violation of causality and the predictive information must have been obtained from the interaction of the retina with the past or current stimulus input. However, this simple version of predictive behavior, that is, carrying non-zero predictive information, is not in itself impressive as it can be exhibited by a passive sensor with or without a delay. Generally, we can expect the mutual information between the output of a passive sensor and its stimulus input to peak at the current time or with a lag (negative δ*t*) that corresponds the processing or propagation time of the system. To produce an output that is more informative of the stimulus at a targeted time in the future than at the current moment requires the system to filter out variability that is more pertinent to the current stimulus but has less bearing at the targeted time in the future. And, this stronger version of predictive behavior is what we discovered to be exhibited by the retina. Presumably, this predictive capability is implemented in retina through a population of cells and their specially wired circuitries. However, we did not perform experiments to determine the cell types explicitly. According to Schwartz et al. (2007) and Palmer et al. (2015), the OSR and predictive behaviors of the retina are not restrictive to certain cell types. Also, from our experimental evidence of OSR-test (see Figure S1, Supplementary information), it is very likely that the recorded channels are dominated by OFF-sustain ganglion cells. It would be important to understand how biological systems can implement this predictive behavior through different response types, retinal circuitries, and physiological mechanisms.

Finally, we would like to point out that, in our experiments, incoming information is coded into time intervals while we are using firing rates of the retina to compute the mutual information between the input and the response. This coding strategy is consistent with the dependency on pulse intervals of firing rate in OSR (Schwartz and Berry, 2008). However, this is probably why the mutual information obtained from experiments is always <5% of the incoming signal. A comparable quantification could possibly be obtained from alternative coding strategies such as considering spike configurations of a population of cells. In the case of our “gedanken” retina, we can extract a much higher amount of information because the coding is known. Note also that the shift of the peak in Figure 6 (gedanken retina) is not proportional to the number of steps *N* for the targeted future. There seem to be a maximum shift in the peak position even for very large *N*. Presumably, this maximum of shift of peak position is controlled both by the stochastic nature of the input signals and the predictive mechanism of Equations (1) and (2). For a real retina, information about this predictive mechanism can be revealed by this maximum time shift of the peak of the *I*_*m*_(δ*t*) curve. For moving stimuli, it is relatively known that neural field models (Mi et al., 2016) or cascade model with feedback control (Berry et al., 1999) for a retina can successfully produce the anticipative tracking of a moving object spatially. This implies that the peak of *I*_*m*_(δ*t*) curve could also be maximized at a positive time shift for a stochastic moving bar. It is still less well understood how such an active process is produced in the time domain. Knowledge of this mechanism should be helpful for the understanding of this anticipative dynamics from the physical structure of the networks in the retina.

## Ethics statement

This study was carried out in accordance with the recommendations of animal protocol, Institutional Animal Care and Use Committee of Academia Sinica (IACUC, AS). The protocol was approved by the Institutional Animal Care and Use Committee of Academia Sinica.

## Author contributions

KSC: Experiments and data analysis, CCC: Computation Modeling and data analysis; CKC: Article planning and writing.

### Conflict of interest statement

The authors declare that the research was conducted in the absence of any commercial or financial relationships that could be construed as a potential conflict of interest.
