# Supplementary material for: Characterization of Predictive Behavior of a Retina by Mutual Information
Source: Front Comput Neurosci. 2017 Jul 20;11:66. doi: 10.3389/fncom.2017.00066 (PMC5517465; doi:10.3389/fncom.2017.00066)
Supplement: Supplementary file 1 [file Presentation1.PDF]

Supplementary Material: Characterization of Predictive Behavior of a Retina by Mutual Information

Kevin Sean Chen Chun-Chung Chen * and C. K. Chan *

*Correspondence:
Chun-Chung Chen
cjj@phys.sinica.edu.tw

C. K. Chan
ckchan@gate.sinica.edu.tw

1 SUPPLEMENTARY DATA

1.1 OSR

We probe the omitted stimulus response (OSR) in the bullfrog retina by repeating periodic light stimuli and varying the period. The LED light source and the square wave pulse duration for stimulation are the same as those used in the main text. The same stimulus consists of 20 pulses (inter-pulse intervals between 100 to 200 ms and duration of 50 ms) is repeated for 10–20 trials with 3–5 s inter-trial resting time. As shown in Figure S1a-c, different firing patterns are observed at different channels of the multi-electrode array (MEA) under the same periodic input, and the OSR is synchronized between multiple channels after the termination of the stimulus. Firing rate of the OSR is usually higher than the response of the retina to each probe pulse, which can even be silent in some channels.

In our experiments, we find that the relation between the period of the stimulus and the relative latency from the last pulse to the OSR is linear within a certain range. As shown in Figure S1d, the linear relation is observed approximately when the period is in the range between 100 and 250 ms and the timing of OSR is most synchronized (reflected by the small error bar in the figure) when the period is around 180 ms. This dynamic range of OSR differs from other species reported previously, but is consistent over all bullfrog retina samples (Schwartz et al., 2007; Schwartz and Berry, 2008).

1.2 Information Measurements

As mentioned in the main text, measured mutual information is corrected for the limited-sampling effects by applying methods of Strong et al. (Strong et al., 1998). We sub-sample from the series of S and R by 5 different fractions f, where $1/f$ is 1–5, and randomly repeat each sub-sampling for 10 times. The measured bias I_{bias} in bits from each sub-sampling is plotted against the inverse of the fraction, $1/f$, and fitted to a quadratic form:

$$I_{\text{bias}} = I_{\text{correct}} + \frac{a}{L} + \frac{b}{L^2}, \tag{S1}$$

where a and b are the fitted coefficients and L is the length of sub-sampled data. The corrected information content I_{correct} is then estimated by extrapolating the values when the data size approaches infinite. Figure S3 shows an example of how the information content scales with the sub-sampled fraction in our measurements.

In the main text, mutual information $I_m(S, R, \delta t)$ was calculated by separating the pulse intervals of stimulation S into 25 states and using spike counts of the response R in bins of the size 50 ms. To verify the validity of this choice of parameters, we have also used different number of states and binning times to calculate the peak of $I_m(S, R, \delta t)$ and its location δt_p. It is shown in Figure S3 and Figure S4 that changing these parameters does not affect the measured values significantly. For the number of states, both the peak values of $I_m(S, R, \delta t)$ and its position (δt_p) are not affected if the number of states is larger than 15. For the binning time, we find that the peak value of $I_m(S, R, \delta t)$ and δt_p also remain relatively unchanged when the window is smaller than 200 ms.

1.3 LN model

In order to compare our results with the conventional understanding for retinal firing patterns, the linear-nonlinear (LN) model (Chichilnisky, 2001) is also tested. The LN model assumes that the firing rate of retinal ganglion cells can be derived from a filtered stimulus passing through a static nonlinear function. In our experiments, the linear part is obtained by reverse correlation under a spatially uniform binary white noise stimulus with a time step of 50 ms, continuing for 3 min. Typically, more than 1000 spikes are recorded from a sorted channel within this duration, and the resulting temporal spike-triggered average (STA) curve would be smooth enough for further analysis. This stimulation is provided by the same light-emitting diode (LED) light source used for stochastic light pulses, ensuring the identical intensity, contrast and wavelength. The temporal kernel $f(t)$ for each selected cell is estimated by interpolation with cubic spline function. The signal convoluted with the stimulation s is then:

$$z(t) = \int_0^t f(t-\tau)\, s(\tau)\, d\tau \tag{S2}$$

The nonlinearity is then fitted with the values of the linear prediction $z(t)$ and the actual firing rate $r(t)$ from our recordings with $r(t) = \sigma(z(t))$, where σ is the logistic function:

$$\sigma(z) = \frac{1}{1 + e^{-(\beta_0 - \beta_1 z)}}, \tag{S3}$$

where β_0 and β_1 are coefficients fitted in the logistic function. The logistic regression is performed through general linear model fitting function provided in MATLAB (glmfit). For statistic significances, nonlinear functions are applied when p-values of the regression are less than 0.05.

The resulting STA and logistic function are shown in Figure S6a-b. As shown in Figure S6c, the LN model captures the recorded temporal firing patterns under flickering stimulation. Note that the rescaling of firing rate in LN model would not affect the result since we use the same binning and partition methods as in the experimental data to obtain $I_m(\delta t)$. More than half of the STA forms are biphasic under spatially uniform flickering LED stimuli, and the time course spans approximately for 400–600 ms. However, neither monotonic nor biphasic STA forms or different time scales reproduce the experimental results. This simplified model describes most of the firing patterns under a flickering stimulus but fails to capture the mutual information curves, as described in the main text.

We estimate the firing rate under stochastic pulse intervals with the fitted LN model. Figure S7 is the result obtained from such a procedure compared with the experiments. It can be seen that the LN model fails to capture the asymmetry observed in the experiments and over estimates the response delay. Regardless to the varying statistics of the stimulation, the resulted peak of $I_m(\delta t)$ from LN model is approximately fixed at the time delay similar to the effective time course of its linear filter (≈400 ms). Similar to the failure to

produce OSR (Gao et al., 2009) and prediction for bar motion (Palmer et al., 2015) with LN model, this implies that an active process or additional mechanism is required to compensate for the response delay and meet the experimental results.

REFERENCES

Chichilnisky, E. J. (2001). A simple white noise analysis of neuronal light responses. *Network (Bristol, England)* 12, 199–213. doi:10.1080/net.12.2.199.213

Gao, J., Schwartz, G., Berry, M. J., and Holmes, P. (2009). An oscillatory circuit underlying the detection of disruptions in temporally-periodic patterns. *Network: Computation in Neural Systems* 20, 106–135. doi:10.1080/09548980902991705

Palmer, S. E., Marre, O., Berry, M. J., and Bialek, W. (2015). Predictive information in a sensory population. *Proceedings of the National Academy of Sciences* 112, 6908–6913. doi:10.1073/pnas.1506855112

Schwartz, G. and Berry, M. J., 2nd (2008). Sophisticated temporal pattern recognition in retinal ganglion cells. *Journal of Neurophysiology* 99, 1787–1798. doi:10.1152/jn.01025.2007

Schwartz, G., Harris, R., Shrom, D., and Berry, M. J. (2007). Detection and prediction of periodic patterns by the retina. *Nature Neuroscience* 10, 552–554. doi:10.1038/nn1887

Strong, S. P., Koberle, R., van Steveninck, R. R. d. R., and Bialek, W. (1998). Entropy and information in neural spike trains. *Physical Review Letters* 80, 197–200. doi:10.1103/PhysRevLett.80.197

2 SUPPLEMENTARY TABLES AND FIGURES

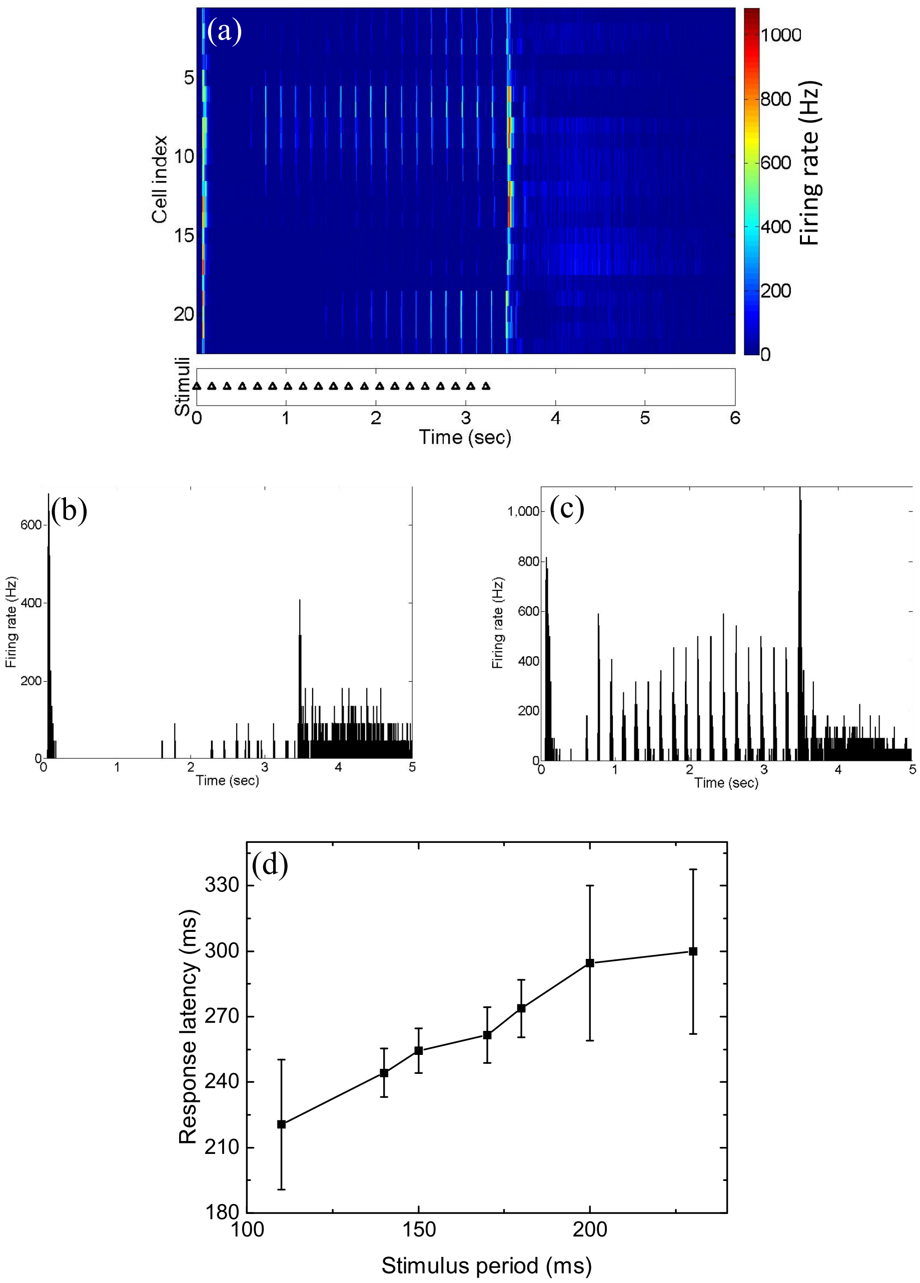

Figure S1. OSR recorded in the bullfrog retina. (a) Raster plot for 22 recorded units under stimuli with period=170ms. (b-c) PSTH of number 9 and 15 cells shown in (a). (d) Response latency of the OSR to the last pulse under different periodic stimuli. Error bars reflecting deviation between the OSR latencies from 8 selected cells. Note that the sustained firing patterns after the stimulation indicates the OFF sustain response type, which is predominant in out recordings.

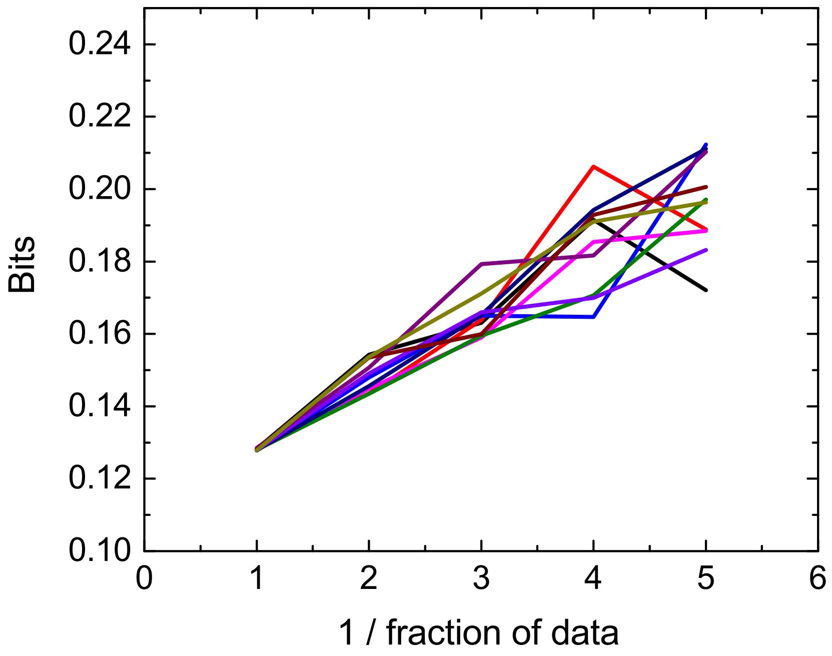

Figure S2. Mutual information calculated under different sub-sampled fractions for a stimulation with $\langle\tau\rangle = 200$ ms and $\tau_{cor} = 2$ s. This example shows 10 random sub-sampling (different colors) from one recorded channel with time shift $\delta t = 0$. For this example, the extrapolated value is approximately 0.11 bits. This corresponds to ∼2.2 bits/sec, since the binning size is 50 ms.

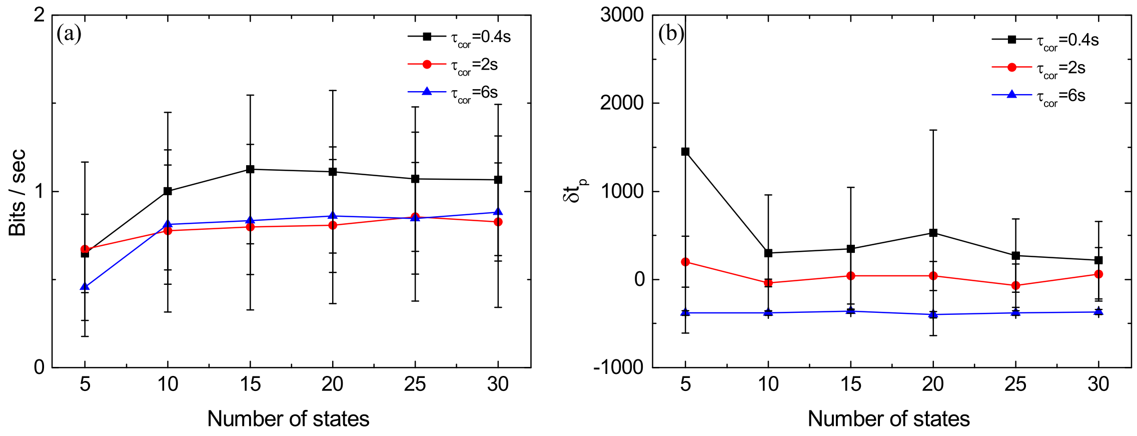

Figure S3. Number of states dependence of measured characteristics of $I_m(S, R, \delta t)$: (a) Peak value of $I_m(S, R, \delta t)$ and (b) peak position (δt_p); measured with partitioning of stimulation into different number of states. Stimulations with fixed $\langle\tau\rangle = 200$ ms and three different τ_{cor} are used. The error bars indicate one standard deviation from 5 randomly selected channels.

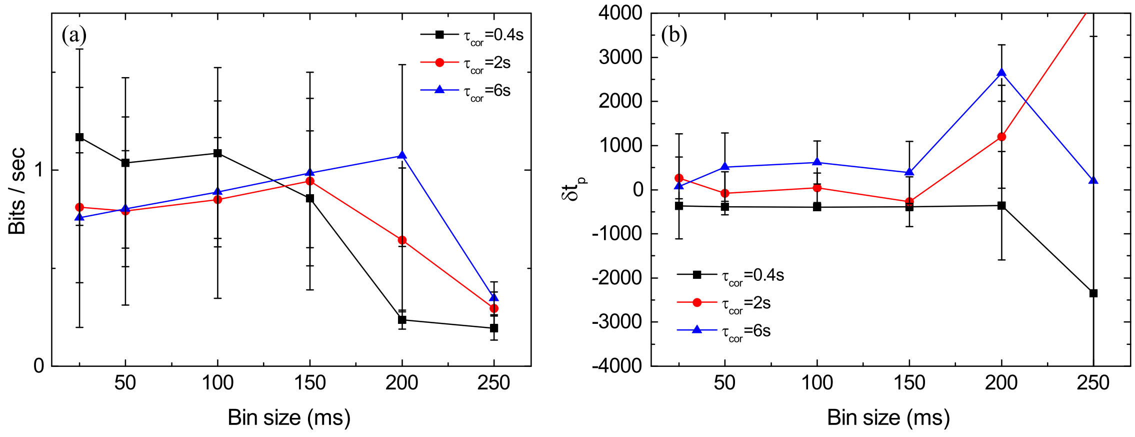

Figure S4. Bin-size dependence of measured characteristics of $I_m(S, R, \delta t)$: (a) Peak value of $I_m(S, R, \delta t)$ and (b) peak position (δt_p); measured with different binning window size for the firing rate R. Stimulations with fixed $\langle \tau \rangle = 200ms$ and three different τ_{cor} are used. The error bars indicate one standard deviation from 5 randomly selected channels.

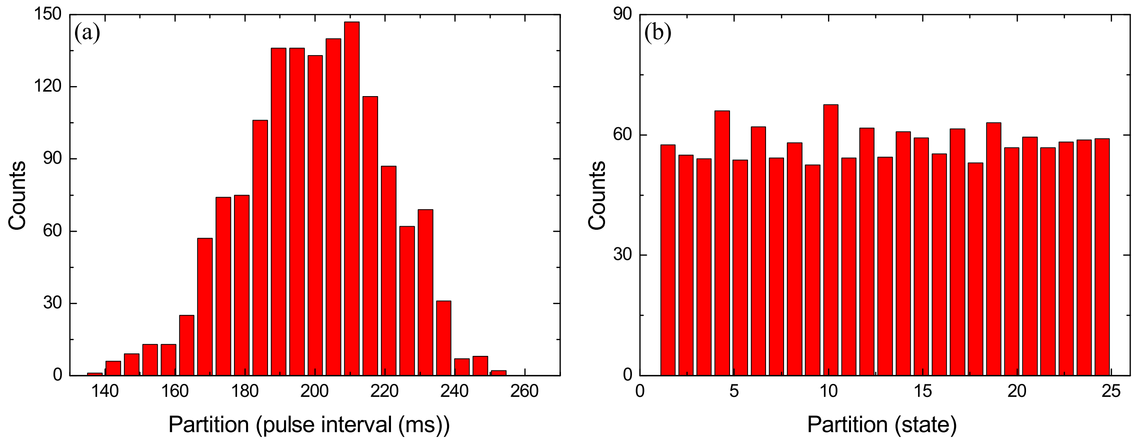

Figure S5. (a) The original distribution of the pulse interval generated from HMM. Histogram plotted in bins with equal size. (b) The approximately equally distributed states calculated from the same stimuli in (a), by changing the bins adaptively. Note that using two different kinds of state distributions do not affect our conclusion from the mutual information measurements.

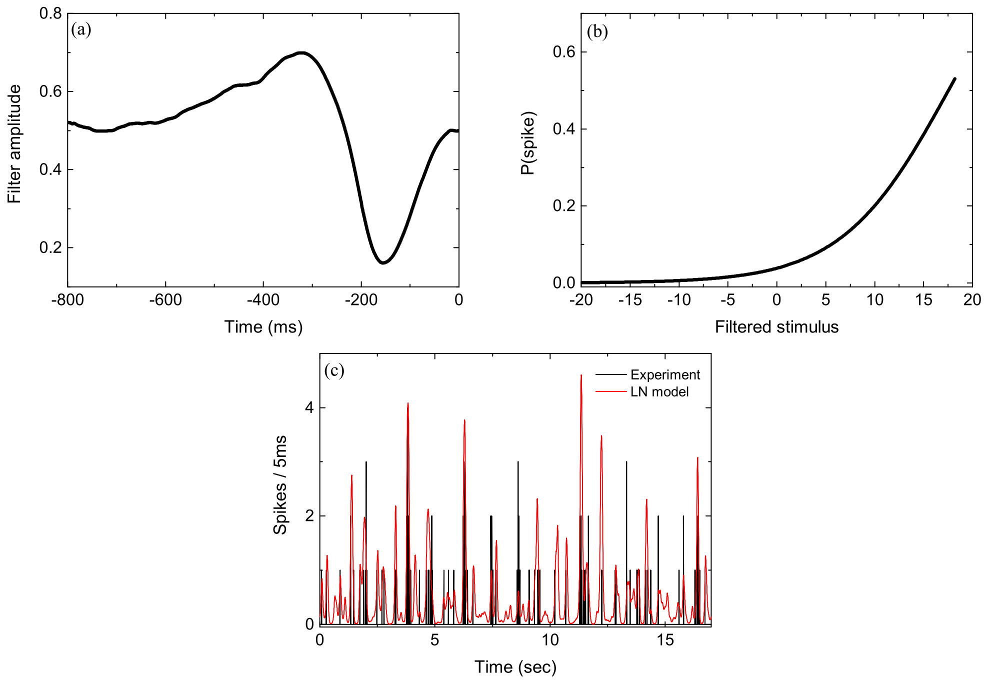

Figure S6. LN model for bullfrog retina. (a) The biphasic STA obtained by reverse correlation. (b) Nonlinearity that controls the firing rate. (c) Comparison between recorded spiking patterns from experiment and fitting by LN model under flickering LED stimulation.

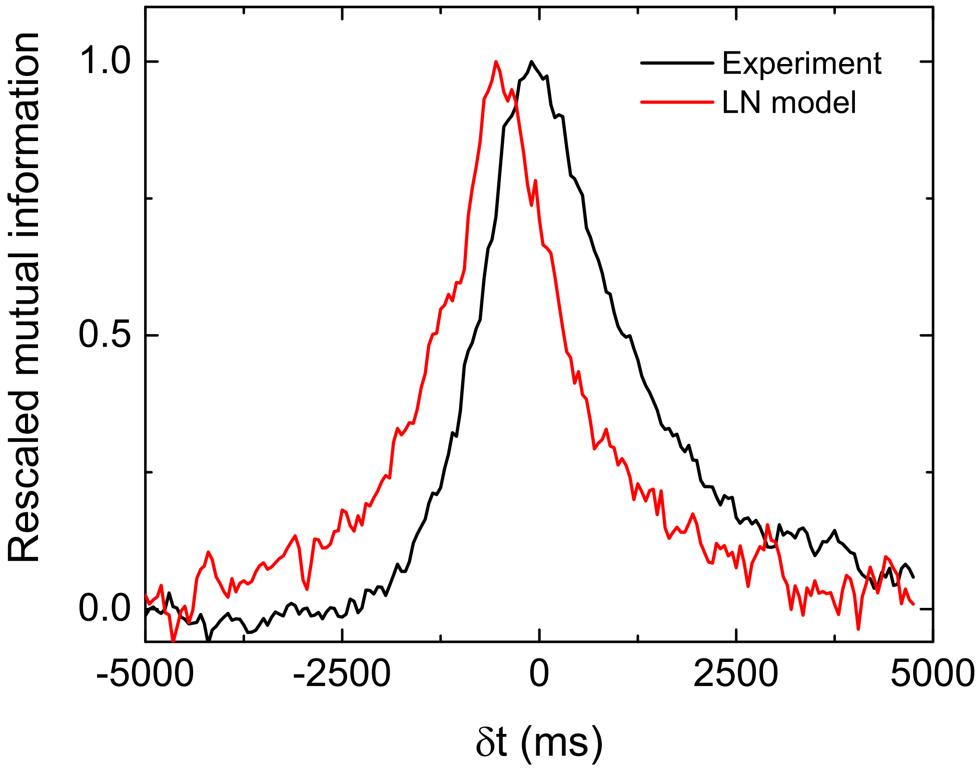

Figure S7. Comparison of $I_m(\delta t)$ with modeled response. The experimental result (with parameters the same as those in Fig.1 in the main text) is shown in black. The red curve is obtained from the LN model. These $I_m(\delta t)$s are normalized by their peak values for the ease of comparison. See text for details.

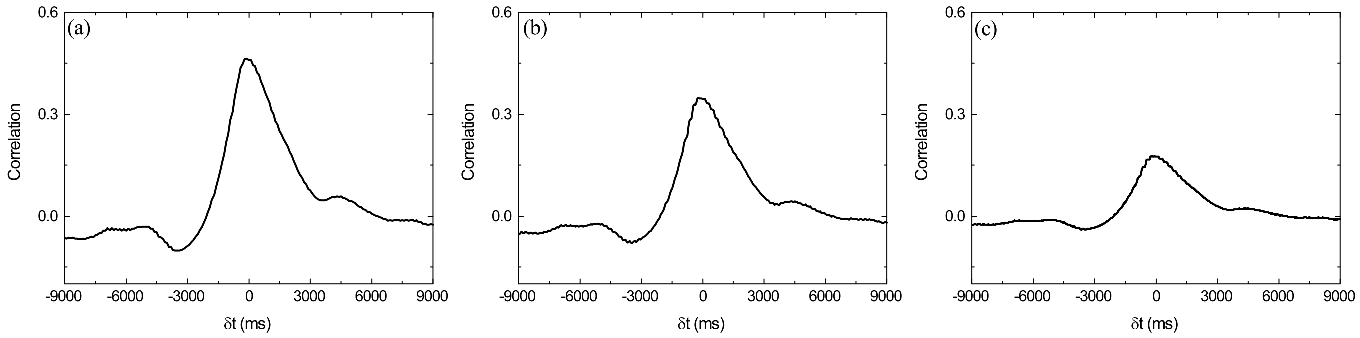

Figure S8. Cross-correlogram calculated between the firing patterns from one channel and the state of pulse intervals (statistics of stimulation is the same as Fig. 1 in the main texts). The bin size is 100 ms in (a), 50 ms in (b), and 5 ms in (c). Note that the position of the peak is unchanged when the precision is narrowed down to a smaller bin size. This peak position might correspond to δt_p in the I_m curves in the main text, but the correlation coefficient is affected significantly by the bin size.
